# Malaria and COVID-19 coinfection in a non-malaria-endemic area in Brazil

**DOI:** 10.1590/0037-8682-0598-2022

**Published:** 2023-05-22

**Authors:** Verônica Diniz Rocha, Larissa W Brasil, Erika de Oliveira Gomes, Ricardo Khouri, Gilcivaldo de Jesus Ferreira, Beatriz Vasconcelos, Marcela de Sá Gouveia, Thais Souza Santos, Mitermayer G Reis, Marcus Vinícius Guimarães Lacerda

**Affiliations:** 1 Instituto Couto Maia, Salvador, BA, Brasil.; 2 Instituto de Pesquisa Clínica Carlos Borborema, Fundação de Medicina Tropical Dr. Heitor Vieira Dourado, Manaus, AM, Brasil.; 3 Instituto Leônidas & Maria Deane, Fiocruz, Manaus, AM, Brasil.; 4 Universidade do Estado do Amazonas, Manaus, AM, Brasil.; 5 Instituto Nacional de Pesquisas da Amazônia, Manaus, AM, Brasil.; 6 Instituto Gonçalo Moniz, Fundação Oswaldo Cruz, Salvador, BA, Brasil.; 7 Universidade Federal da Bahia, Hospital Universitário Professor Edgard Santos, Salvador, BA, Brasil.; 8 Universidade Federal da Bahia, Faculdade de Medicina, Salvador, BA, Brasil.; 9 Yale School of Public Health, Yale University, New Haven, Connecticut, United States .; 10 Universidade Federal da Bahia, Salvador, BA, Brasil.

**Keywords:** Malaria, COVID-19, SARS-CoV-2

## Abstract

Differential diagnosis of coronavirus disease 2019 (COVID-19) from other febrile diseases is one of several challenges imposed by the pandemic. We present a case of severe malaria and COVID-19 coinfection in a non-malaria-endemic region. A 44-year-old female with malaise, fever, hypotension, jaundice, and enlarged liver and spleen was admitted to the intensive care unit. Reverse transcription-quantitative PCR results for severe acute respiratory syndrome coronavirus 2 were positive. Rapid tests, microscopy, and quantitative PCR were positive for *Plasmodium vivax*. Cytokine storm profiles were identified. We could not determine whether the severe vivax malaria in our patient was triggered by COVID-19 coinfection.

## INTRODUCTION

In 2021, Brazil registered 139,211 malaria cases, predominantly in the Brazilian Amazon^1^. Some areas, such as indigenous, rural, settlements, and mining regions, are frequently associated with a greater risk of malaria transmission, with rural areas responsible for 41.2% of the total autochthonous cases in the Brazilian Amazon. Approximately 99.9% of malaria transmission in Brazil occurs in the Amazon region, with 33 municipalities accounting for 80.0% of the total autochthonous malaria cases in 2021. In the extra-Amazonian region, 62.9% of reported cases occurred in patients who had visited endemic areas, deemed “imported” cases^1^.

The coronavirus disease 2019 (COVID-19) pandemic has imposed several challenges, including the differential diagnosis from other febrile diseases^2^. Throughout the COVID-19 pandemic, rural areas experienced infections later than urban regions. In rural regions, particularly the northern part of Brazil with a high incidence of malaria, a high burden of COVID-19 combined with poor healthcare capacities may have enhanced the morbidity and mortality of both diseases or even impaired adequate diagnosis in some settings^3^.

A brief search of Google Scholar, PubMed, and Lilacs using the terms “malaria” and “COVID-19” identified several case reports, case series, cohorts and systematic reviews; however, none addressed cytokine production in cases of malaria and COVID-19 coinfection. The first case of malaria and COVID-19 coinfection was reported in June 2020 in Qatar, presenting a patient who had traveled to Pakistan three months prior to hospitalization. The patient was a young male without comorbidities, with severe *Plasmodium vivax* malaria, and it was suggested that the case was a reactivation of a previous infection with dormant liver stage parasites or hypnozoites during the COVID-19 infection^4^. In Brazil, only one case of coinfection was reported in a healthy male who had traveled to a rural area in the state of Mato Grosso^5^. 

Herein, we present a case of severe *P. vivax* malaria and COVID-19 coinfection in a rural settlement in the extreme south of Bahia (Brazil), a non-malaria-endemic region. In addition to describing the clinical case, unlike previous reports, we examined the cytokine profile of cytokines to understand the pathophysiology underlying this coinfection.

## CASE REPORT

In June 2021, a 7-year-old male without comorbidities from the south of Bahia (northeastern Brazil), residing in a peri-urban area, reported fever, chills, fatigue, headache, myalgia, dyspnea, pallor, apathy, and reduced food intake. Other family members reported similar symptoms. The patient was transferred to a reference hospital for infectious diseases in Salvador/Bahia. The patient presented with pallor, tachycardia, and tachypnea without signs of respiratory distress. A physical examination revealed painless hepatomegaly and splenomegaly. Laboratory tests identified hemoglobin 6.2 g/dL, hematocrit 17.4%, leukocytes 5,820/mm^3^ and platelets 50,000/µL. The severe acute respiratory syndrome coronavirus 2 (SARS-CoV-2) IgM rapid test result was positive, although the nasal swab SARS-CoV-2 reverse transcription-quantitative PCR (RT-qPCR) result was negative. 

Although a non-malaria-endemic region, a recent malaria outbreak was confirmed in a rural settlement neighboring the region where the patient and his family resided. A rapid test for malaria was positive for *P. vivax,* confirmed by microscopy and quantitative PCR (qPCR) after genomic DNA extraction from slides (Supplementary Figure 1). A thin blood-smear examination identified 378.3 parasites/µL of blood, parasitemia of half a cross. The patient was initially treated with three doses of intravenous artesunate 2.4 mg/kg/dose every 12 h, then two doses of 2.4 mg/kg/dose daily, followed by oral chloroquine with primaquine, resulting in full recovery.

During the child’s hospitalization, his mother, a 44-year-old female with mild hypertension, complained of malaise and fever, receiving treatment at the emergency department of the same hospital. On admission, she presented with a ten-day history of fever, chills, headache, myalgia, asthenia, and weight loss. She was diagnosed with hypotension (95×70 mmHg) in the emergency room, which resolved following intravenous rehydration. She was admitted to the adult intensive care unit without oxygen support or vasoactive drugs. Physical examination revealed mild jaundice and an enlarged liver and spleen. No changes in the respiratory or cardiac systems were observed.

Laboratory tests on admission revealed hemoglobin 10.1 g/dL; hematocrit 27.5%; leukocytes 4,670/mm^3^ (segmented 79%); platelets 47,000/µL; INR 1.0; C-reactive protein 151.2; total bilirubin 2.0 mg/dL (direct bilirubin 1.2 mg/dL); gamma-glutamyl transferase 86 U/L; blood urea nitrogen (BUN) 53 mg/dL; creatinine 1.6 mg/dL; glutamic-oxaloacetic transaminase 38 U/L; aspartate aminotransferase 18 U/L; sodium 125 mEq/L, potassium 2.6 mEq/L; creatine phosphokinase (CPK) 188 U/L; lactate dehydrogenase 854 U/L. The SARS-CoV-2 RT-qPCR results were positive with a nasal swab. Chest computed tomography revealed small ground-glass opacities with less than 5% involvement and laminar pleural effusion.

**FIGURE 1:**
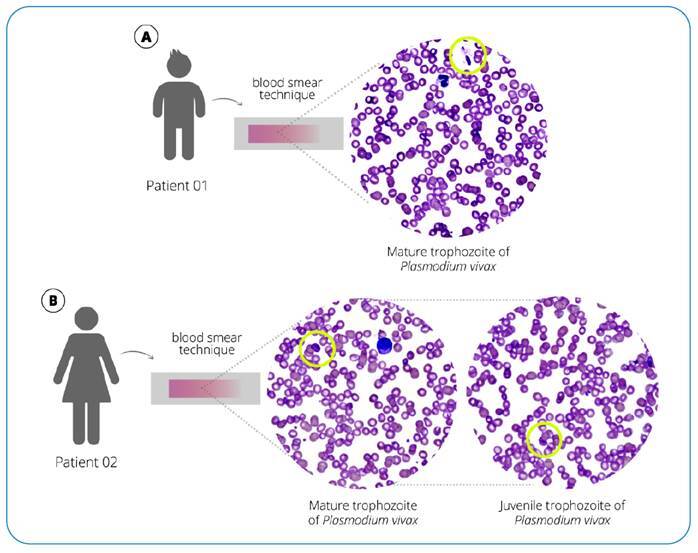
Trophozoites of *Plasmodium vivax* in peripheral blood, using Giemsa-stained blood smear observed on an Easy Scan^®^ Thermo Scientific automated microscope. **A.** Case 1 - child **B.** Case 2 - mother.

**FIGURE 3:**
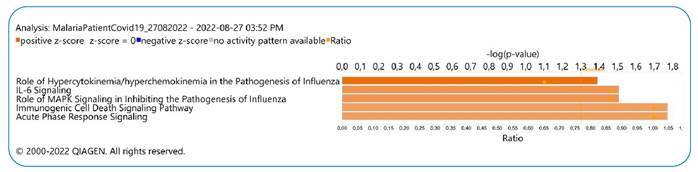
Respiratory infection pathogenesis canonical pathway enrichment analysis in plasma sample of *Plasmodium vivax* and SARS-CoV-2 co-infected-patient (mother). (A) Top 5 canonical pathways with Fisher’s exact test p value <0.05 and absolute Z-score >2.

*P. vivax* was identified using a rapid malaria test and confirmed by microscopy and qPCR after genomic DNA extraction from slides ( Supplementary Figure 1). A thin blood-smear examination identified 2078.15 parasites/µL of blood, parasitemia of two crosses. (Figure 1). A cytokine storm profile was identified ( Supplementary Information 2) (Figures 2 and 3). 

Plasma levels of 29 cytokines were measured in *P. vivax* and SARS-CoV-2-co-infected patients (mother), *P. vivax*-infected patient (child), and healthy donors (control). Expression levels of interleukin (IL)-6, IL-10, granulocyte colony-stimulating factor, IL-1RA, MIP-1a, interferon (IFN)-γ, tumor necrosis factor (TNF)-α, MIP-1b, IL-8, IL-1b, IP-10, IL-1a, IL-7, IL-13 were increased by ˃10-fold in the co-infected patient (mother) when compared with mean cytokine levels of the healthy donor and *Plasmodium vivax*-infected patient (child) (Figure 2); these findings are compatible with the cytokine storm signature of severe respiratory infection pathogenesis, as revealed by biological enrichment analysis using IPA software (https://digitalinsights.qiagen.com/IPA (Figure 3). 

**FIGURE:**
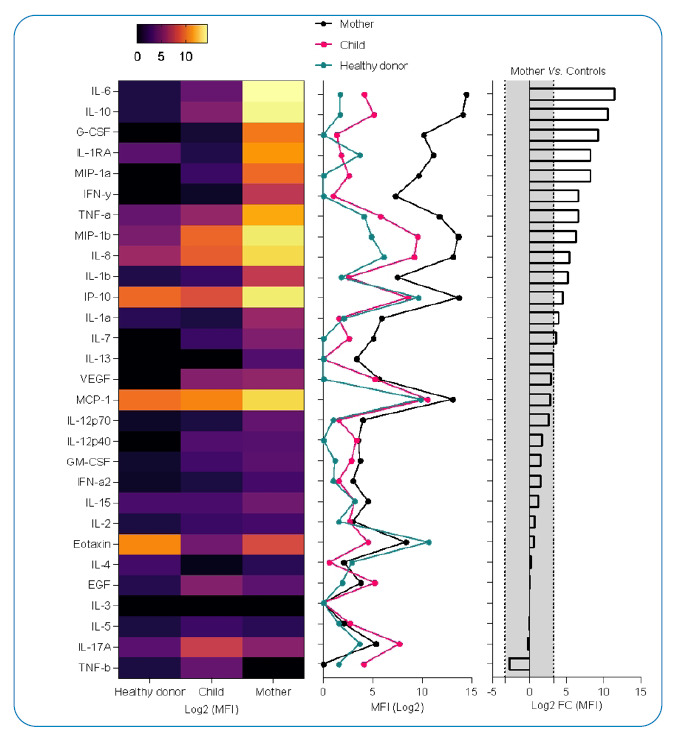
2: Cytokine storm signature in the plasma sample of a *Plasmodium vivax* and SARS-CoV-2 co-infected-patient (mother). (A) Heatmap showing log2 of median fluorescent intensity (MFI) levels. (B) Line plot showing log2 of MFI levels. (C) Bar plot showing log2 of fold change calculation of MFI levels of *P. vivax* and SARS-CoV-2 co-infected-patient (mother) *vs.* mean of MFI levels of the healthy donor and *P. vivax-*infected patient (child); the gray area represents a <10-fold change absolute value. EGF: epidermal growth factor; G-CSF: granulocyte colony-stimulating factor; GM-CSF: granulocyte-macrophage colony-stimulating factor; IL: interleukin; IFN: interferon; MCP-1: monocyte chemoattractant protein-1; TNF-a,-b: tumor necrosis factor-α, -β; VEGF: vascular endothelial growth factor.

Treatment with intravenous artesunate (2.4 mg/kg/dose at 0, 12, and 24 h) was initiated. After administering three parenteral doses and following parasitemia of ˂1%, treatment was changed to oral hydroxychloroquine 600 mg/day for three days and primaquine 30 mg/day for seven days. The patient subsequently recovered completely. 

The Research Ethics and Committee approved the publication of this work.

## DISCUSSION

Herein, we report a case of *P. vivax* malaria and COVID-19 coinfection, a severe form of vivax malaria in a 44-year-old female with mild hypertension without evidence of severe manifestations of COVID-19. Despite the malarial diagnosis and high probability of SARS-CoV-2 infection through contact with parents, COVID-19 could not be established in the child, given the possibility of a false-positive SARS-CoV-2 result via a rapid serological test. SARS-CoV-2 RT-PCR was performed ten days after the first symptoms were noted in the child, which might explain the possible false-negative result. Similar to the mother, the child’s father also had a positive SARS-CoV-2 RT-PCR result, increasing the possibility of a false-negative SARS-CoV-2 RT-PCR result in the child. Following the arrival of an individual from the Brazilian Amazon with untreated malaria, malaria cases were documented in a rural settlement near an urban region.

Endemic diseases, including malaria, can be difficult to distinguish from COVID-19, given similarities in symptoms, and the same difficulty can occur in cases of malaria and COVID-19 coinfection, as described in previous case reports^4^ ^,^ ^5^. A retrospective cohort study in Sudan enrolled 321 confirmed patients with COVID-19 and 270 patients with malaria co-infected with COVID-19. Compared with SARS-CoV-2 monoinfection, coinfection with COVID-19 and malaria was associated with increased all-cause in-hospital mortality^6^. Coinfection with COVID-19 and severe malaria has been reported previously, although poorly explored. To the best of our knowledge, our study is the first to describe the cytokine profile in a patient with severe malaria infection who was COVID-19 positive^7^ ^,^ ^8^. Herein, the cytokine storm identified in the co-infected patient may explain the trend toward high clinical severity in malaria and COVID-19 co-infected patients described in previous studies. 

During COVID-19 infection, monocytes and macrophages are increased, elevating levels of proinflammatory cytokines, including IL-6, IL-1, TNF-α, and IL-8, which in some patients causes a cytokine storm, resulting in *acute respiratory distress syndrome* and multiple organ failure^9^. Patients with severe SARS-CoV or Middle East respiratory syndrome coronavirus (MERS-CoV) infections reportedly exhibit significantly higher serum levels of IL-6, IFN-α, CCL5, CXCL8, and CXCL-10 than patients with milder infections^10^. Although the patient’s mother had malaria and COVID-19 coinfection, she did not have a severe form of COVID-19; she experienced a cytokine storm, which may be related to the occurrence of severe malaria.

In individuals with asymptomatic *P. vivax* infection, levels of circulating pro- and anti-inflammatory cytokines do not increase, and a cytokine storm is not observed. Excessive innate immune cell activation and the resulting cytokine storm are responsible for the initial signs and symptoms of malaria and mediate severe forms of the disease. During the acute phase of *P. vivax* infection, high levels of proinflammatory cytokines, including IL-1β, IL-6, TNFα, IL-8, and IL-10 (an anti-inflammatory cytokine), are consistently expressed in symptomatic patients. Excessive innate immune cell activation can lead to a cytokine storm with adverse effects on the host^11^. 

We could not establish whether the severe vivax malaria observed in our patient was triggered by COVID-19 coinfection. Several resource-limited regions worldwide experienced challenges performing confirmatory tests for COVID-19, with some deemed malaria endemic. Some symptoms of malaria and COVID-19 overlap, making it possible for co-infected patients to receive a diagnosis of only one of the diseases, compromising the patient’s treatment and control of these diseases in the community^12^.
