# Supplementary material for: Malaria and COVID-19 coinfection in a non-malaria-endemic area in Brazil
Source: Rev Soc Bras Med Trop. 2023 May 22;56:e0598-2022. doi: 10.1590/0037-8682-0598-2022 (PMC10204151; doi:10.1590/0037-8682-0598-2022)
Supplement: Supplementary file 1 [file 1678-9849-rsbmt-56-e0598-2022-supp1.pdf]

SUPPLEMENTARY MATERIAL

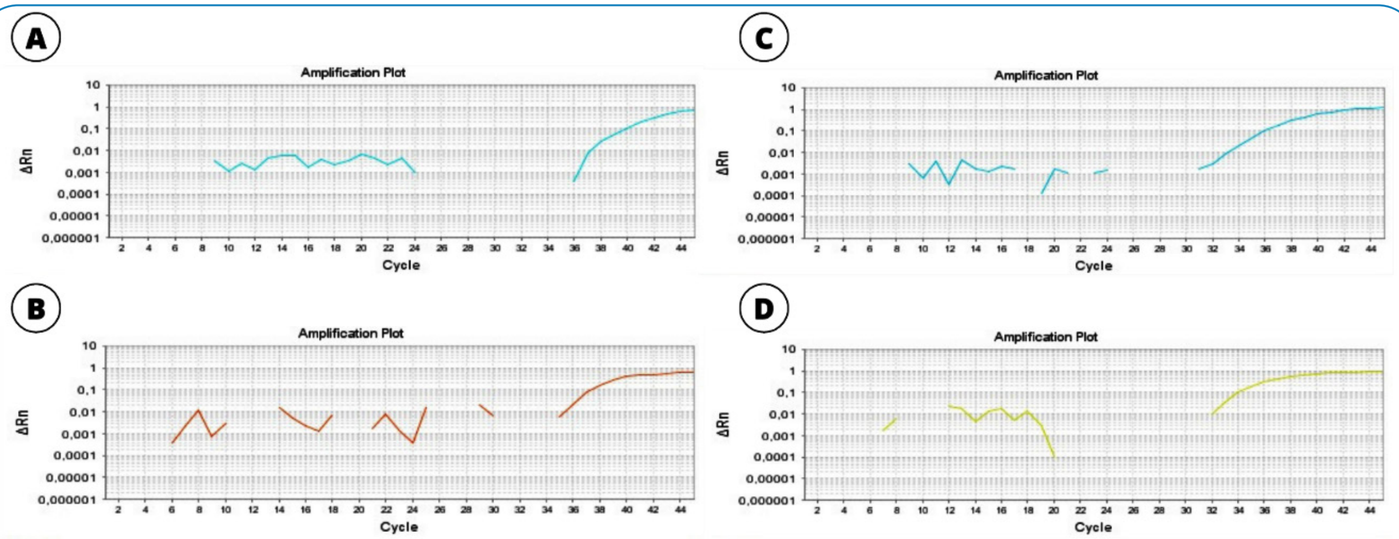

SUPPLEMENTARY FIGURE 1: Real-time qPCR of patient 01 and patient 02. **A.** Amplification using a gene-specific primer of *Plasmodium* spp. of patient 01. **B.** Amplification using a species-specific primer of *Plasmodium vivax* of patient 01. **C.** Amplification using a gene-specific primer of *Plasmodium* spp. of patient 02. **D.** Amplification using a species-specific primer of *Plasmodium vivax* of patient 02.

OPEN ACCESS
