# Supplementary material for: Malaria and COVID-19 coinfection in a non-malaria-endemic area in Brazil
Source: Rev Soc Bras Med Trop. 2023 May 22;56:e0598-2022. doi: 10.1590/0037-8682-0598-2022 (PMC10204151; doi:10.1590/0037-8682-0598-2022)
Supplement: Supplementary file 2 [file 1678-9849-rsbmt-56-e0598-2022-supp2.pdf]

SUPPLEMENTARY INFORMATION 2

Materials and Methods

Quantification of soluble markers in the plasma

The concentrations of the following soluble markers (CCL2/MCP-1, CCL3/MIP-1α, CCL4/MIP-1β, CXCL8/IL-8, CXCL10/IP-10, EGF, Eotaxin, G- CSF, GM-CSF, IFN-α2, IFN-γ, IL-10, IL-12P40, IL-12P70, IL-13, IL-15, L-17A, IL-1RA, IL-1α, IL-1β, IL-2, IL-3, IL-4, IL-5, IL-6, IL-7, TNF-α, TNF-β, VEGF) in plasma (kept frozen at −80°C) were measured using MILLIPLEX MAP Human Cytokine/Chemokine Magnetic Bead Panel 29-Plex (Merck Millipore, USA), following the manufacturer's instructions.

Canonical pathway enrichment analysis

IPA software was used for enrichment analysis to identify relevant biological canonical pathways (QIAGEN Inc., https://www.qiagenbioinformatics.com/products/ingenuity-pathway-analysis). Briefly, the canonical pathway enrichment analysis p-value calculation was based on the reference set of 29 measured cytokines, and a cutoff >10-fold change was considered statistically significant.

cc BY OPEN ACCESS
